# Faster growth of the major prokaryotic versus eukaryotic CO_2_ fixers in the oligotrophic ocean

**DOI:** 10.1038/ncomms4776

**Published:** 2014-04-29

**Authors:** Mikhail V. Zubkov

**Affiliations:** 1 Ocean Biogeochemistry & Ecosystems Research Group, National Oceanography Centre, European Way, Southampton SO14 3ZH, UK

## Abstract

Because maintenance of non-scalable cellular components—membranes and chromosomes—requires an increasing fraction of energy as cell size decreases, miniaturization comes at a considerable energetic cost for a phytoplanktonic cell. Consequently, if eukaryotes can use their superior energetic resources to acquire nutrients with more or even similar efficiency compared with prokaryotes, larger unicellular eukaryotes should be able to achieve higher growth rates than smaller cyanobacteria. Here, to test this hypothesis, we directly compare the intrinsic growth rates of phototrophic prokaryotes and eukaryotes from the equatorial to temperate South Atlantic using an original flow cytometric ^14^CO_2_-tracer approach. At the ocean basin scale, cyanobacteria double their biomass twice as frequently as the picoeukaryotes indicating that the prokaryotes are faster growing CO_2_ fixers, better adapted to phototrophic living in the oligotrophic open ocean—the most extensive biome on Earth.

## Abstract (web summary)

After the energetically superior eukaryotes had evolved, prokaryotes appeared to lose control over biological CO_2_ fixation in all major biomes on Earth. Here the author shows that in the oligotrophic ocean, the most extensive biome on Earth, the prokaryotes fix CO_2_ twice as fast as eukaryotes.

## Graphical abstract

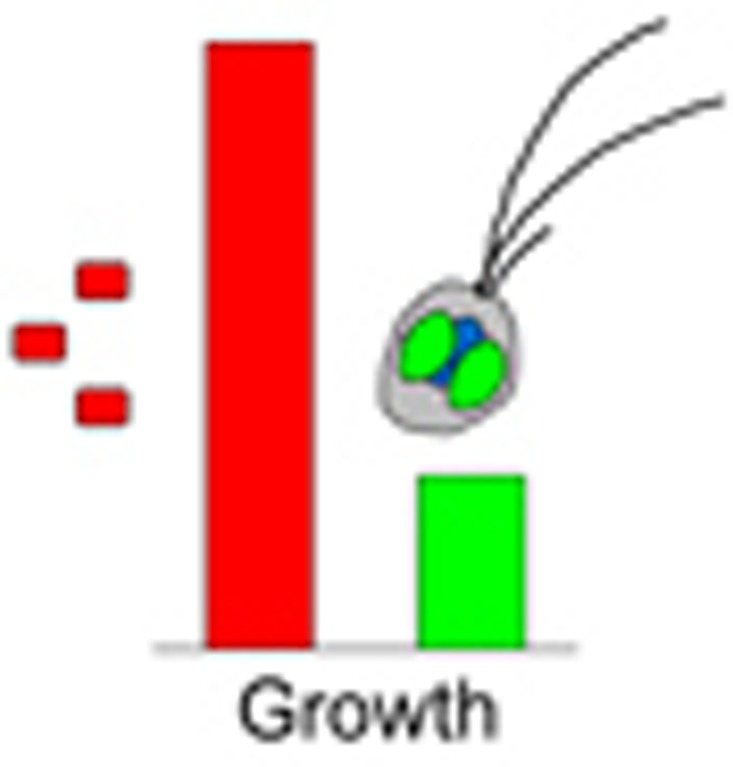

Since the beginning of life on Earth, prokaryotes have regulated the main global biogeochemical cycles^1^,^2^. However, after the energetically superior eukaryotes^3^ had evolved, prokaryotes appeared to lose control over biological CO_2_ fixation in all major biomes including the open ocean. Apparently non-scalable cellular components such as chromosomes and membranes handicap growth rates of the smaller phytoplankton cells (cell diameter ~1 μm) relative to larger cells (cell diameter 4–8 μm)^4^,^5^. There is supporting field evidence that phototrophic picoeukaryotes dominate over cyanobacteria in primary production in coastal^6^ and certain oceanic regions^7^,^8^. On the other hand the above handicap could be counterbalanced by nutrient transport efficiency, which decreases with increase in cell size^9^,^10^.

Direct comparison of *in situ* growth rates of phototrophic prokaryotes and eukaryotes in the oceanic subtropical gyres can show whether prokaryotes or picoeukaryotes are best adapted to oligotrophic conditions. Compared with other oceanic biomes these gyres are seasonally relatively stable and have remained steady for the geological time necessary for biological co-evolution of these organisms to occur. Because biomass growth of both groups primarily depends on photosynthesis, it is justifiable to compare their growth using the CO_2_ fixation rates. Until now, cellular biomass of oceanic microbes has been calculated from biovolume, determined microscopically^8^, or using a change in impedance^6^ or by size fractionation^11^, and multiplied by a poorly constrained volume-to-carbon conversion factor, derived from culture studies^12^, under an assumption that the factor is unaffected by growth conditions.

Here, to circumvent the above assumption, a ^14^C-tracer experimental method, in which cumulative cellular CO_2_ fixation was determined at the end of the light period (12 h) and at the end of the following 12-h dark period (24 h), is developed to compare growth and biomass of the smallest oceanic plastidic protists and cyanobacteria. We find that cyanobacteria grow twice as fast as the smallest eukaryotes both in the South Atlantic gyre and in adjacent regions. Despite their protracted co-evolution simple prokaryotic CO_2_ fixers outgrow complex eukaryotic CO_2_ fixers in the oligotrophic gyre ecosystems highlighting the under-rated growth capacity of phototrophic prokaryotes.

## Results

### Prokaryotic and eukaryotic groups examined for CO_2_ fixation

Sixteen experiments were completed in the South Atlantic Ocean in three oceanic regions: the Equatorial convergence region (EQ), Southern subtropical gyre (SG) and Southern temperate region (ST) (Supplementary Fig. 1) to compare representative mean growth rates that should encompass natural variations in cell abundance and nutrient availability. The following cells were flow sorted: *Prochlorococcus* (*Pro*)^13^, *Synechococcus* (*Syn*), small plastidic protists (Pl-S, cell diameter ~2 μm), large plastidic protists (Pl-L, cell diameter ~3 μm)^8^,^14^,^15^. In addition, low-nucleic acid (LNA)-containing bacterioplankton and aplastidic protists (A-Pl, cell diameter ~3 μm) were flow sorted as controls.

To compare cellular growth and biomass of the smallest oceanic plastidic protists and cyanobacteria, ^14^CO_2_ fixation by flow cytometrically enumerated and sorted *Pro*, *Syn*, Pl-S and Pl-L cells was measured at the end of the light period (12 h) and at the end of the following 12-h dark period (24 h). The amount of tracer in flow-sorted *Pro*, *Syn*, Pl-S and Pl-L cells was generally higher at 12 h than at 24 h, while the opposite was true for A-Pl cells (Supplementary Figs 2 and 3). The amount of tracer in flow-sorted LNA cells was statistically indistinguishable from the background (Supplementary Fig. 3), indicating that the SAR11 alphaproteobacteria, which comprised the LNA cell population^13^, did not fix CO_2_. The increased ^14^C-radioactivity of the A-Pl cells in the dark has a ready explanation—the A-Pl cells continued feeding on ^14^C-labelled CO_2_ fixers in the dark. The decreased ^14^C-radioactivity of the *Pro*, *Syn*, Pl-S and Pl-L cells in the dark (Supplementary Fig. 2b–f) can depend on two cellular processes. First, the cells had directly lost carbon fixed during the light period (primarily by respiration but also exudation, egestion or lysis). Second, the reduction in cellular radioactivity was a result of cell division, because smaller daughter cells would contain less tracer than the larger parent cells and therefore an increased proportion of daughter cells will decrease average radioactivity per cell in the sampled population.

### Dark respiration versus cell division

To assess the magnitude of dark physiological loss of ^14^C, tracer retention by each population as well as by their sum was compared at 12 and 24 h (Supplementary Fig. 4). Average cellular ^14^C content was multiplied by corresponding cell concentrations (Supplementary Fig. 5). A statistically significant increase in radioactivity of the A-Pl population between 12 and 24 h (Supplementary Fig. 4b) was a result of A-Pl cells feeding on ^14^C-labelled phototrophic cells in the dark. No statistically significant difference in the *Pro*, Pl-S and Pl-L populations was found between the two time points indicating that dark respiration of ^14^C-labelled material in those cells was insignificant. Statistical significance of the decrease in the radioactivity of the *Syn* population (Supplementary Fig. 4e) was more likely due to grazing on *Syn* cells by A-Pl, mixotrophic Pl-S and Pl-L cells^14^,^15^ than to *Syn* dark respiration, because the ^14^CO_2_ fixed at 12 and 24 h by the sum of all flow-sorted populations (including predators) was similar (Supplementary Fig. 4a).

The above negligibly small dark respiration of ^14^C-labelled cellular material is in agreement with earlier laboratory experiments on algal cultures^16^ with dark respiration being 2–16% of daily fixed CO_2_. A 12% value was calculated for nitrate-limited chemostat-growing cultures of *Monochrysis lutheri*^17^, when washout from a chemostat was taken into account. Relative dark respiration for a batch culture of *Isochrysis galbana* grown from nutrient-replete into nutrient-starved conditions was about 4%^18^. For *Skeletonema costatum* grown under nutrient-replete conditions at photon flux densities of 50 and 1,200 μmol photons m^−2^ s^−1^ dark respiration was 2% and 16% of photosynthesis, respectively^19^. However, there were also reports of considerable dark respiration that accounted for 30–40% of daylight CO_2_ fixation in oligotrophic waters^20^ and 20–25% of daylight CO_2_ fixation in the temperate North Atlantic^21^. The discrepancy between the reported high dark respiration and the presented data (Supplementary Fig. 4) presumably originated from the methodological differences. The published high values were obtained by gently collecting live phytoplanktons on filters. In the present study, phytoplankton samples were fixed to preserve cell integrity for subsequent flow sorting. Only ^14^C tracer incorporated into macromolecules remained in fixed cells and ^14^C tracer incorporated into labile cellular material was released on fixation, whereas labelled labile material remained in the unfixed cells^22^. Labile material such as sugars is preferentially respired by cells^23^, while turnover of labelled macromolecules in cells is slow^24^. In the present study, the dark respiration of macromolecules was examined and found negligibly low (Supplementary Fig. 4), and hence cell division was considered as the main process responsible for radiotracer decrease in mean cells in the dark (Supplementary Fig. 2).

*Pro* and *Syn* as well as the smallest plastidic protists synchronize their cell division after dusk^25^,^26^,^27^,^28^,^29^ (M.V.Z., unpublished observations). Therefore assumption of night cell division is plausible. This means that only the difference between divisions in the first 12 light hours and the subsequent 12 dark hours can be determined. Accordingly, the derived growth rates are conservative estimates.

### Group-specific growth rates

The observed increases in concentrations of cells between 12 and 24 h (Supplementary Fig. 5) were used to independently assess group-specific growth rates for comparison with the rates determined using the ^14^C tracer method. The growth rates of the Pl-S and Pl-L, estimated by the two methods, were statistically similar (Supplementary Fig. 6), proving that the tracer method gave realistic estimates of growth rates of plastidic protists.

The tracer-derived growth rates of the *Pro* and *Syn* were 2–4 times higher than their cell-derived growth rates. This discrepancy can be reconciled if cyanobacteria either have high rates of dark respiration, or are under strong mortality pressure. In the first case, mean dark respiration should be >50% for *Pro* and *Syn* in the SG+EQ and EQ, respectively, and 80% for *Syn* in the ST. These high values are unlikely (see above, Supplementary Fig. 4e,f). The increased radioactivity of the A-Pl in the dark (Supplementary Figs 2a and 4b), and similar radioactivity of the sum (Supplementary Fig. 4a) support grazing on cyanobacteria by the A-Pl, Pl-L and Pl-S. Those grazing-related ^14^C retentions would reduce the tracer-based estimates of growth rates of Pl-S and Pl-L. Indeed, their tracer-derived growth rates showed a trend of being lower than their cell-derived growth rates (Supplementary Fig. 6); however, the differences were statistically insignificant, suggesting that phototrophic CO_2_ fixation is the main contributor to the growth of Pl-L and Pl-S cells. Accordingly, grazing being the main cause of the difference between the tracer-derived and cell-derived growth rates of the *Pro* and *Syn* (Supplementary Fig. 6), the former estimated the intrinsic growth of cyanobacteria, whereas the latter estimated the net growth of cyanobacteria.

### Comparison of the growth rates of the CO_2_ fixers

**Figure 1:**
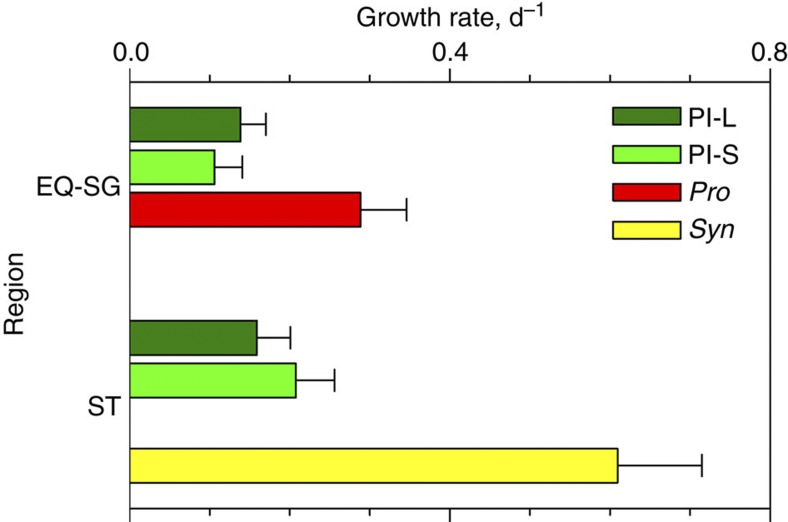
Relative growth rates of phytoplankton groups. Comparison of the growth rates of large plastidic protists (Pl-L), small plastidic protists (Pl-S), *Prochlorococcus* (*Pro*) and *Synechococcus* (*Syn*) in the two regions: combined Equatorial waters (EQ) and Southern gyre (SG) and Southern temperate waters (ST). Error bars indicate propagated single s.e.

The region-averaged growth rates of the studied groups ranged between 0.1 and 0.6 d^−1^ (Fig. 1). The *Syn* grew fastest in the EQ regions, 0.7 d^−1^; however, these data were omitted from the figure as unrepresentative for the combined EQ+SG regions, because *Syn* growth was not determined in the SG region owing to their low cell concentrations (Supplementary Fig. 1b). The growth rates of *Pro* in the EQ (0.3–0.6 d^−1^) are similar to *Pro* growth rates determined at the base of the photic layer in the Sargasso Sea^30^ and in agreement with rates of up to one doubling per day, determined in the equatorial Pacific using cell cycle analysis^25^. The mean growth rates of *Syn* in the ST and *Pro* in the EQ+SG were, respectively, three and two times higher than the mean growth rates of Pl-L or Pl-S (Supplementary Fig. 6). Therefore, the growth rates of both cyanobacteria in all regions studied were significantly higher than the growth rates of the smallest plastidic protists.

The finding that plastidic protists have lower growth rates than cyanobacteria (Fig. 1) requires reconciliation with the hypothesis of optimal cell size to achieve maximum photosynthetic growth^4^,^5^. In the present study, the smallest eukaryotes showed growth rates (Fig. 1; Supplementary Fig. 6) of one half or less compared with the growth rates of eukaryotes of comparable size in nutrient-rich culture^5^. If the taxon-independent approach is applied, the above suggests that eukaryotes are underperforming in the oligotrophic open ocean, being poorly adapted to their habitat. An alternative explanation could be that the eukaryotes cultured in the laboratory are physiologically different from the eukaryotes inhabiting the open ocean, for example, the latter are mixotrophs under-represented in culture collections but capable of outcompeting obligate phototrophs in nature^31^. If this is correct, the difference between potential and realized growth rates is as likely to be related to physiological divergence as to evolutionary inadequacy.

**Figure 2:**
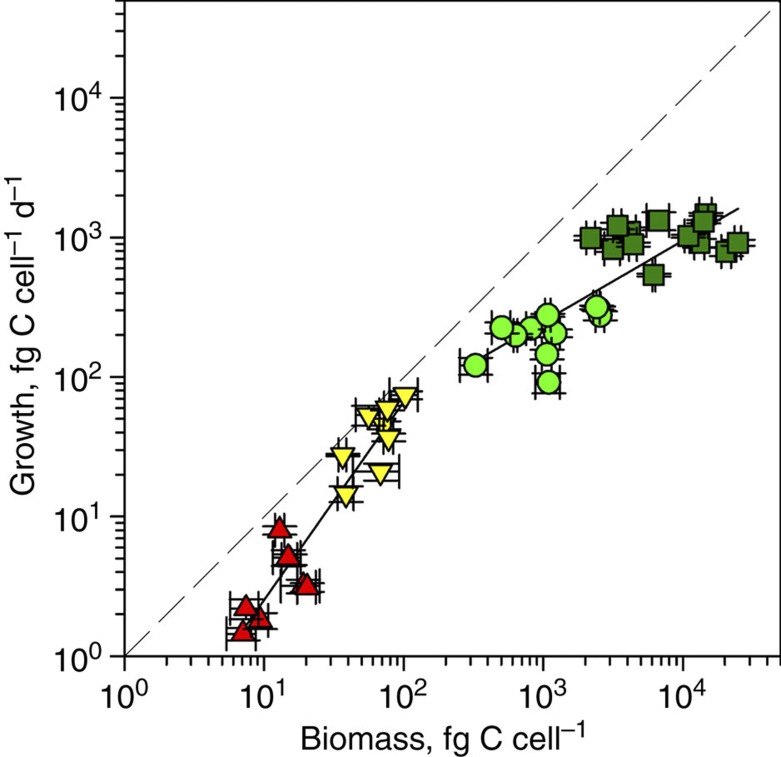
Metabolic scaling in prokaryotes and eukaryotes. Scatter plot of biomass versus daily growth of *Prochlorococcus* (red triangles), *Synechococcus* (yellow triangles), small plastidic protists (green circles) and large plastidic protists (green squares) to compare their growth. Solid lines indicate linear regressions of cyanobacteria (slope=1.4, *r*^2^=0.89, *P*<0.001) and picoeukaryotes (slope=0.6, *r*^2^=0.67, *P*<0.001). Dashed line indicates equality of biomass and daily growth.

There is more controversy about the *in situ* growth rates of the *Pro* and *Syn* in the EQ (Figs 1 and 2; Supplementary Fig. 6) being about twice as high as their growth rates in the nutrient unlimited culture at similar light intensity^5^. Because the *in situ* rates cannot be higher than the maximum rates, it is probable that either low temperature of 18 °C or high nutrient conditions in culture experiments were less optimal for the cyanobacterial growth than the low nutrient conditions in the open ocean.

### Fixation rates and biomasses of the CO_2_ fixers

**Figure 3:**
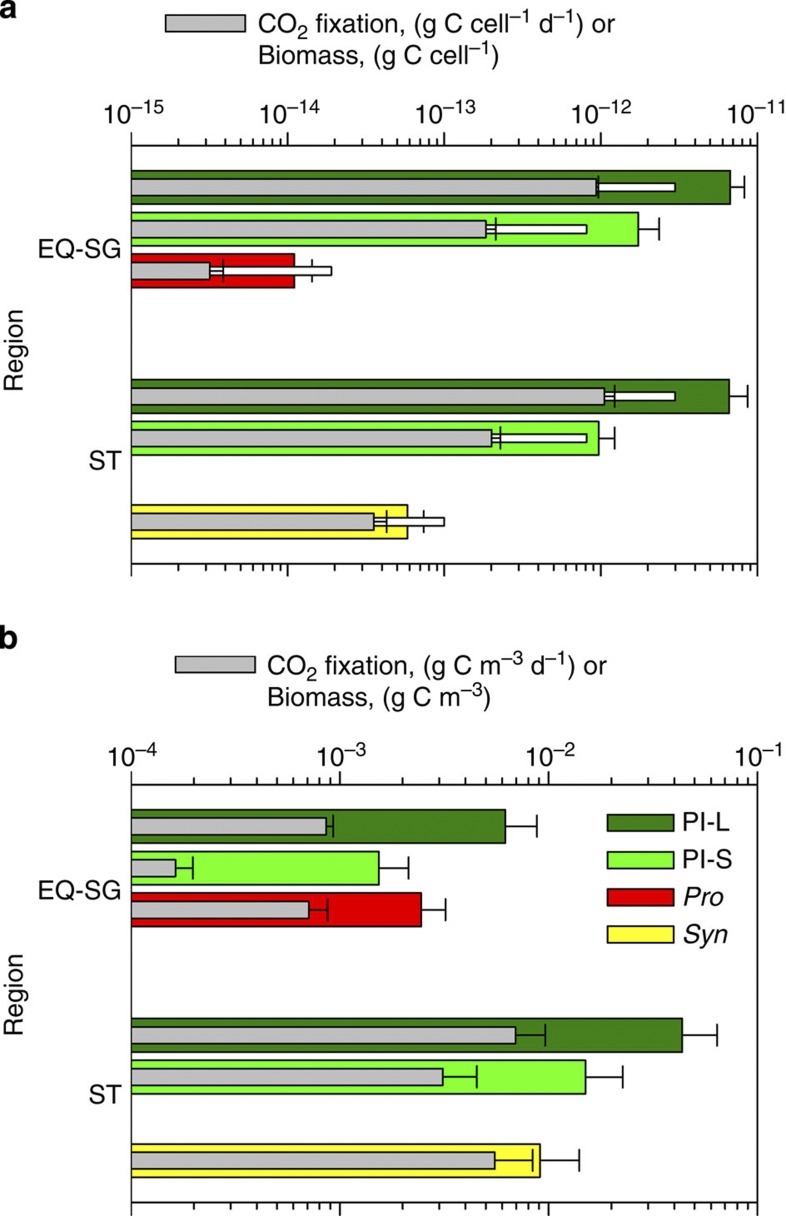
Absolute biomasses and growth rates of phytoplankton groups. Comparison of the ^14^C tracer-based estimates of cellular (**a**) and population (**b**) biomasses and growth rates (internal intermediate grey bars) of large plastidic protists (Pl-L), small plastidic protists (Pl-S), *Prochlorococcus* (*Pro*) and *Synechococcus* (*Syn*) in the two regions: combined Equatorial waters (EQ) and Southern gyre (SG) and Southern temperate waters (ST). Thin white bars (**a**) indicate biovolume-based estimates of cellular biomass. Note the log scale of the *x* axis. Error bars indicate propagated single s.e.

The cellular CO_2_ fixation rates of the studied groups span four orders of magnitude in direct proportion to their cellular biomasses (Fig. 3a). The average fixation rates of the Pl-L and Pl-S cells were statistically similar in all regions and the average fixation rates of the *Syn* cells were comparable in the EQ and ST regions. Being more sensitive to nutrient conditions, the cellular CO_2_ fixation rates of cyanobacteria were more variable than the rates of eukaryotes (Supplementary Fig. 2).

The average biomass of *Pro* and *Syn* cells was 60% lower and the biomass of Pl-S and Pl-L was 20-120% higher according to the tracer method compared with the biovolume method^11^,^15^ (Fig. 3a). First, it was reassuring that the estimates based on the two independent approaches were comparable. Second, because *Pro* and *Syn* cell diameters were estimated by size fractionation using broad-stepped (0.2 μm) pore size filters it was unsurprising that the biovolume-based method gave higher biomass estimates. Third, the microscopic size measurements of fixed and consequently shrunk Pl-S and Pl-L cells using nucleic acid-specific stain would underestimate the living cell sizes. Hence, cellular biomass, estimated using the sensitive ^14^C-tracer method, is more likely to be closer to the real biomass of living cells. Among the studied groups *Pro* cells were the smallest (Fig. 3a). Taking a 1.7-Mbp genome size of *Pro* MED4 strain for guidance^32^, the *Pro* chromosome would constitute 5% of cellular biomass, which is within the theoretical limits^4^ for a viable phototrophic cell.

Within the limited size range of the studied microbes a relationship between the cellular biomass and biomass growth rate indicates a slope difference between the cyanobacteria and smallest plastidic protists (Fig. 2). The slope drop from prokaryotes to eukaryotes is in agreement with the step shift during a transition from heterotrophic prokaryotes to protozoa^33^, suggesting similarity in bioenergetic scaling between phototrophic and heterotrophic microbes. Such similarity could be linked to commonness of versatile metabolism among oceanic CO_2_-fixing microbes—mixotrophy of the smallest eukaryotes^15^ and photoheterotrophy of cyanobacteria^13^.

At the population level the biomasses of the *Pro* and *Syn* were, respectively, 40% and 20% of the biomass of the Pl-L (Fig. 3b), or merely 25% of the sum of Pl-L, Pl-S and *Pro* in the EQ+SG and 13% of Pl-L, Pl-S and *Syn* in the ST. Daily CO_2_ fixation by the *Pro* and *Syn* populations equated to 80% of the fixation by the Pl-L population, or 40 and 35% by the corresponding sums. Although eukaryotes clearly dominated the biomass of the CO_2_ fixers in the studied regions, cyanobacterial populations fixed CO_2_ more effectively and grew faster. If grazing control by eukaryotes was relaxed, cyanobacterial populations could outgrow populations of plastidic protists within days because of faster cyanobacterial reproduction.

## Discussion

It seems that after >10^9^ years of co-evolution^34^ the smallest phototrophic eukaryotes have not succeeded in exceeding the growth of cyanobacteria in the oligotrophic open ocean. However, if, in addition to competing for major inorganic nutrients, eukaryotes feed on bacteria^14^,^35^,^36^, then eukaryote survival as predators would depend on growth of phototrophic prokaryotes. The nutritional adaptations of photrophic eukaryotes and prokaryotes in the nutrient-depleted open ocean have established, perhaps, the simplest ecosystem of faster growing photoheterotrophic prokaryotes largely controlled by mixotrophic eukaryotes.

## Methods

### Sampling

The experimental study was carried out on board the UK Royal Research Ship *James Cook* during the Atlantic Meridional Transect (AMT) cruise number JC079 (AMT22) in October–November 2012. Seawater samples were collected before dawn with 20-litre Niskin bottles mounted on the sampling rosette of a conductivity-temperature-depth (CTD) profiler (Sea-Bird Electronics, Washington, USA) from a depth of 20 m, a representative depth of the surface mixed layer unaffected by the ship’s movement and contamination. Samples were gently transferred into a 10-l polypropylene carboy, before being dispensed through a spigot into 120-ml Pyrex glass bottles. All plastic and glasswares were soaked in 10% HCl and extensively rinsed with sampled seawater. All experiments were set up within 20 min after sample collection.

### CO_2_ fixation experiments

To assess the effect of high NaH^14^CO_3_ tracer addition, necessary to determine CO_2_ fixation rates by the tiniest phototrophic prokaryote—*Pro* cyanobacteria^30^, radiotracers of two manufacturers were used. Sodium bicarbonate as a solution (Perkin-Elmer, USA) was added directly to sampled seawater, while crystals of sodium bicarbonate (Hartmann Analytic, Germany), were dissolved in autoclaved seawater, collected from 20 m depth (the latter tracer gave on average 12% higher estimates of CO_2_ fixation than the former one—data not shown). Working solutions of both radiotracers were filtered through a 0.2-μm pore size filter before being added to sampled seawater (90–240 kBq ml^−1^ final radioactivity). The bottled samples were then incubated at ambient temperatures controlled by a refrigerated water bath (Grant Instruments, UK) in a 6-l acrylic glass water tank illuminated by a warm white light-emitting diode array (Photon Systems Instruments, Czech Republic) adjusted to 350 μmol photons m^−2^ s^−1^, mimicking the average *in situ* light conditions at 20 m depth in subtropical waters. The constant light output was used to allow direct comparison of cellular CO_2_ fixation determined in experiments with seawater samples collected at different latitudes. After 12 h incubation in the light, to mimic the day period, 60 ml of sample were poured into a polypropylene bottle containing 3 ml of 20% paraformaldehyde (PFA, final concentration 1%). PFA solution was prepared by dissolving PFA powder in seawater by stirring on a heating plate followed by filtering of cooled solution through a 0.2-μm pore size filter. The Pyrex bottles containing the remaining 60 ml of sample were transferred into a parallel, temperature-controlled, light-tight 6-l tank and incubated for an additional 12 h in the dark at ambient temperature to mimic the night period. At the end of the experiment, the remaining 60 ml of sample were fixed with 1% PFA. CO_2_ fixation in dark controls were <2% of CO_2_ fixation in the 12-h light incubations (for example, 0.18±0.01 mg C m^−3^ d^−1^ in the dark versus 9.68±0.84 mg C m^−3^ d^−1^ in the light at 4°1.8′N, 26°28.2′W).

Subsamples were taken from each polypropylene bottle containing a fixed sample to determine the total CO_2_ fixation and the exact amount of added radiotracer. Three 1.6-ml subsamples were transferred into 2-ml microcentrifuge tubes to determine absolute concentrations of microbial populations (see below) and to flow sort *Pro* cells, when their natural abundance was sufficiently high to sort at a rate of 150–300 cells min^−1^. A single 20-ml subsample was concentrated using a 0.4-μm polycarbonate filter (Nuclepore, Whatman, UK) mounted on a filtration unit (Swinnex, Millipore, USA) using a syringe pump (KD Scientific, USA) at a flow rate of 2.0 ml min^−1^ and used for sorting stained *Pro* and *Syn* cells as well as Pl-S. The remaining sample was concentrated using a 0.6-μm Nuclepore polycarbonate filter in the same way for flow sorting Pl-L and A-Pl. The seawater retained inside the filtration units as well as the filters were transferred into 2-ml microcentrifuge tubes. The unconcentrated subsamples and two concentrated subsamples of the same 1.6-ml final volume, were stained inside the microcentrifuge tubes with SYBR Green I (Sigma-Aldrich, UK) in three potassium citrate buffer^37^,^38^ and flow sorted within 24 h. Stained samples scheduled for sorting were stored in the dark at 4 °C.

### Flow cytometric sorting

Stained microbial cells from different populations were discriminated, enumerated and flow sorted using a FACSCalibur flow cytometer (Becton Dickinson, Oxford, UK). An internal standard of 0.5 and 1.0 μm diameter, multi-fluorescent beads (Polysciences, Germany) was added to each stained sample before analyses to monitor instrument performance and to determine absolute concentrations of microbial populations^38^,^39^.

For each flow-sorted population 4–6 replicates of different cell numbers were sorted (for example, Supplementary Fig. 3). Bacterial and eukaryotic cells were collected on 0.2 and 0.8-μm polycarbonate filters, respectively, using a three-socket filtration unit and prepared for radio-assaying following the same procedure as for total carbon fixation measurements. Using a liquid scintillation counter (Tri-Carb 3100, Perkin Elmer, UK), samples (a sample was counted for 5 min) from each experiment were batch counted 3–4 times on different days. To account for counting variations a mean of these 3–4 counts was calculated before a relative average cellular rate of CO_2_ fixation was computed as a fraction of the amount of radiotracer added to the experimental bottle. To enable comparison of measured cellular CO_2_ fixation rates between experiments, absolute fixation rates were calculated.

CO_2_ concentrations in sampled seawater, derived from the CTD measurements of seawater salinity^40^ were used to convert relative cellular rates of CO_2_ fixation into absolute CO_2_ fixation rates in units of gram carbon fixed by an average cell of the flow-sorted population. The CO_2_ fixation of flow-sorted *Pro*, *Syn*, Pl-S or Pl-L cells was determined experimentally as a mean cellular daily growth rate *C*^fix^_12_ (g C cell^−1^ d^−1^). The CO_2_ fixation *C*^fix^_24_, determined at 24 h, is a mean for the fraction of cells that divided during the dark period (*D*^div^) and the ones that remained undivided. The divided cells double in number but each cell contains a half of the fixed CO_2_. This simple model can be described by the following formula:


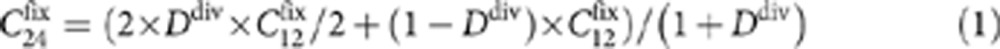


The above formula can be simplified and re-arranged to calculate the daily relative growth rate *D*^div^ [d^−1^] assuming that cells divided during the dark period:


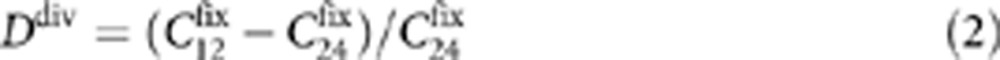


Then the cellular biomass *C*^biom^ (g C cell^−1^) could be computed:


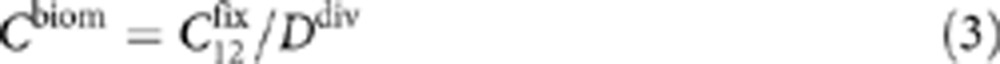


or


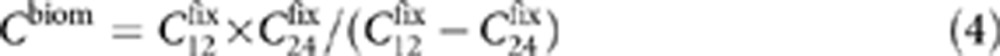


The F-test was used for analyses of variance and the paired *t*-test was used for comparing means or alternatively Wilcoxon’s signed rank test was used in cases when the normality test failed (*P*<0.05). s.e. of mean were propagated when growth rates, biomasses and population-specific CO_2_ fixation rates were calculated.

## Additional information

**How to cite this article:** Zubkov, M. V. Faster growth of the major prokaryotic versus eukaryotic CO_2_ fixers in the oligotrophic ocean. *Nat. Commun.* 5:3776 doi: 10.1038/ncomms4776 (2014).

## Supplementary Material

Supplementary Information Supplementary Figures 1-6

## References

[b1] Kirchman D. L. Carbon cycle—microbial breathing lessons. Nature 385, 121–122 (1997).

[b2] Falkowski P. G., Fenchel T. & Delong E. F. The microbial engines that drive Earth's biogeochemical cycles. Science 320, 1034–1039 (2008). 18497287 10.1126/science.1153213

[b3] Lane N. & Martin W. The energetics of genome complexity. Nature 467, 929–934 (2010). 20962839 10.1038/nature09486

[b4] Raven J. A. Why are there no picoplanktonic O_2_ evolvers with volumes less than 10^−19^ M^3^? J. Plankton Res. 16, 565–580 (1994).

[b5] Maranon E. *et al.* Unimodal size scaling of phytoplankton growth and the size dependence of nutrient uptake and use. Ecol. Lett. 16, 371–379 (2013). 23279624 10.1111/ele.12052

[b6] Worden A. Z., Nolan J. K. & Palenik B. Assessing the dynamics and ecology of marine picophytoplankton: the importance of the eukaryotic component. Limnol. Oceanogr. 49, 168–179 (2004).

[b7] Li W. K. W. Primary production of prochlorophytes, cyanobacteria, and eukaryotic ultraphytoplankton—measurements from flow cytometric sorting. Limnol. Oceanogr. 39, 169–175 (1994).

[b8] Jardillier L., Zubkov M. V., Pearman J. & Scanlan D. J. Significant CO_2_ fixation by small prymnesiophytes in the subtropical and tropical northeast Atlantic Ocean. ISME J. 4, 1180–1192 (2010). 20393575 10.1038/ismej.2010.36

[b9] Chisholm S. W. inPrimary Productivity and Biogeochemical Cycles in the Sea eds Falkowski P. G., Woodhead A. D. 213–237Plenum (1992).

[b10] Banavar J. R., Damuth J., Maritan A. & Rinaldo A. Supply-demand balance and metabolic scaling. Proc. Natl Acad. Sci. USA 99, 10506–10509 (2002). 12149461 10.1073/pnas.162216899 PMC124956

[b11] Zubkov M. V., Sleigh M. A., Burkill P. H. & Leakey R. J. G. Picoplankton community structure on the Atlantic Meridional Transect: a comparison between seasons. Prog. Oceanogr. 45, 369–386 (2000).

[b12] Christian J. R. & Karl D. M. Microbial community structure at the United-States Joint Global Ocean Flux Study Station ALOHA—inverse methods for estimating biochemical indicator ratios. J. Geophys. Res. 99, 14269–14276 (1994).

[b13] Gomez-Pereira P. R. *et al.* Comparable light stimulation of organic nutrient uptake by SAR11 and *Prochlorococcus* in the North Atlantic subtropical gyre. ISME J. 7, 603–614 (2013). 23096403 10.1038/ismej.2012.126 PMC3580278

[b14] Zubkov M. V. & Tarran G. A. High bacterivory by the smallest phytoplankton in the North Atlantic Ocean. Nature 455, 224–226 (2008). 18690208 10.1038/nature07236

[b15] Hartmann M. *et al.* Mixotrophic basis of Atlantic oligotrophic ecosystems. Proc. Natl Acad. Sci. USA 109, 5756–5760 (2012). 22451938 10.1073/pnas.1118179109 PMC3326507

[b16] Geider R. J. & Osborne B. A. Respiration and microalgal growth—a review of the quantitative relationship between dark respiration and growth. New Phytol. 112, 327–341 (1989).

[b17] Laws E. & Caperon J. Carbon and nitrogen metabolism by *Monochrysis lutheri*—measurement of growth rate dependent respiration rates. Mar. Biol. 36, 85–97 (1976).

[b18] Flynn K. J., Davidson K. & Leftley J. W. Carbon-nitrogen relations at whole-cell and free-amino-acid levels during batch growth of *Isochrysis galbana* (Prymnesiophyceae) under conditions of alternating light and dark. Mar. Biol. 118, 229–237 (1994).

[b19] Anning T. *et al.* Photoacclimation in the marine diatom *Skeletonema costatum*. Limnol. Oceanogr. 45, 1807–1817 (2000).

[b20] Geider R. J. inPrimary Productivity and Biogeochemical Cycles in the Sea ed. Falkowski P. 333–360Plenum (1992).

[b21] Marra J. & Barber R. T. Phytoplankton and heterotrophic respiration in the surface layer of the ocean. Geophys. Res. Lett. 31, L09314 (2004).

[b22] Zubkov M. V. & Leakey R. J. G. Evaluation of the efficiency of metabolism of dinoflagellate phosphorus and carbon by a planktonic ciliate. Eur. J. Protistol. 45, 166–173 (2009). 19155165 10.1016/j.ejop.2008.09.003

[b23] Glover H. E. & Morris I. Photosynthetic characteristics of coccoid marine cyanobacteria. Arch. Microbiol. 129, 42–46 (1981).

[b24] Zubkov M. V., Sleigh M. A. & Burkill P. H. Measurement of bacterivory by protists in open ocean waters. FEMS Microbiol. Ecol. 27, 85–102 (1998).

[b25] Vaulot D., Marie D., Olson R. J. & Chisholm S. W. Growth of *Prochlorococcus*, a photosynthetic prokaryote, in the equatorial Pacific Ocean. Science 268, 1480–1482 (1995). 17843668 10.1126/science.268.5216.1480

[b26] Vaulot D. & Marie D. Diel variability of photosynthetic picoplankton in the equatorial Pacific. J. Geophys. Res. 104, 3297–3310 (1999).

[b27] Wyman M. Diel rhythms in ribulose-1,5-bisphosphate carboxylase/oxygenase and glutamine synthetase gene expression in a natural population of marine picoplanktonic cyanobacteria (*Synechococcus* spp.). Appl. Environ. Microbiol. 65, 3651–3659 (1999). 10427062 10.1128/aem.65.8.3651-3659.1999 PMC91547

[b28] Zubkov M. V., Sleigh M. A. & Burkill P. H. Assaying picoplankton distribution by flow cytometry of underway samples collected along a meridional transect across the Atlantic Ocean. Aquat. Microb. Ecol. 21, 13–20 (2000).

[b29] Zubkov M. V. & Quartly G. D. Ultraplankton distribution in surface waters of the Mozambique Channel—flow cytometry and satellite imagery. Aquat. Microb. Ecol. 33, 155–161 (2003).

[b30] Chisholm S. W. *et al.* A novel free-living prochlorophyte abundant in the oceanic euphotic zone. Nature 334, 340–343 (1988).

[b31] Tittel J. *et al.* Mixotrophs combine resource use to outcompete specialists: Implications for aquatic food webs. Proc. Natl Acad. Sci. USA 100, 12776–12781 (2003). 14569026 10.1073/pnas.2130696100 PMC240694

[b32] Rocap G. *et al.* Genome divergence in two *Prochlorococcus* ecotypes reflects oceanic niche differentiation. Nature 424, 1042–1047 (2003). 12917642 10.1038/nature01947

[b33] DeLong J. P., Okie J. G., Moses M. E., Sibly R. M. & Brown J. H. Shifts in metabolic scaling, production, and efficiency across major evolutionary transitions of life. Proc. Natl Acad. Sci. USA 107, 12941–12945 (2010). 20616006 10.1073/pnas.1007783107 PMC2919978

[b34] Knoll A. H., Javaux E. J., Hewitt D. & Cohen P. Eukaryotic organisms in Proterozoic oceans. Philos. Trans. R. Soc. Lond. B Biol. Sci. 361, 1023–1038 (2006). 16754612 10.1098/rstb.2006.1843 PMC1578724

[b35] Frias-Lopez J., Thompson A., Waldbauer J. & Chisholm S. W. Use of stable isotope-labelled cells to identify active grazers of picocyanobacteria in ocean surface waters. Environ. Microbiol. 11, 512–525 (2009). 19196281 10.1111/j.1462-2920.2008.01793.x PMC2702499

[b36] Hartmann M., Zubkov M. V., Scanlan D. J. & Lepere C. In situ interactions between photosynthetic picoeukaryotes and bacterioplankton in the Atlantic Ocean: evidence for mixotrophy. Environ. Microbiol. Rep. 5, 835–840 (2013). 24249292 10.1111/1758-2229.12084

[b37] Marie D., Partensky F., Jacquet S. & Vaulot D. Enumeration and cell cycle analysis of natural populations of marine picoplankton by flow cytometry using the nucleic acid stain SYBR Green I. Appl. Environ. Microbiol. 63, 186–193 (1997). 16535483 10.1128/aem.63.1.186-193.1997 PMC1389098

[b38] Zubkov M. V., Burkill P. H. & Topping J. N. Flow cytometric enumeration of DNA-stained oceanic planktonic protists. J. Plankton Res. 29, 79–86 (2007).

[b39] Zubkov M. V. & Burkill P. H. Syringe pumped high speed flow cytometry of oceanic phytoplankton. Cytomometry A 69, 1010–1019 (2006). 10.1002/cyto.a.20332 16969799

[b40] Parsons T. R., Maita Y. & Lalli C. M. Manual of chemical and biological methods for seawater analysis Pergamon (1984).

