# Supplementary material for: Faster growth of the major prokaryotic versus eukaryotic CO2 fixers in the oligotrophic ocean
Source: Nat Commun. 2014 Apr 29;5:3776. doi: 10.1038/ncomms4776 (PMC4015317; doi:10.1038/ncomms4776)
Supplement: Supplementary Information — Supplementary Figures 1-6 [file ncomms4776-s1.pdf]

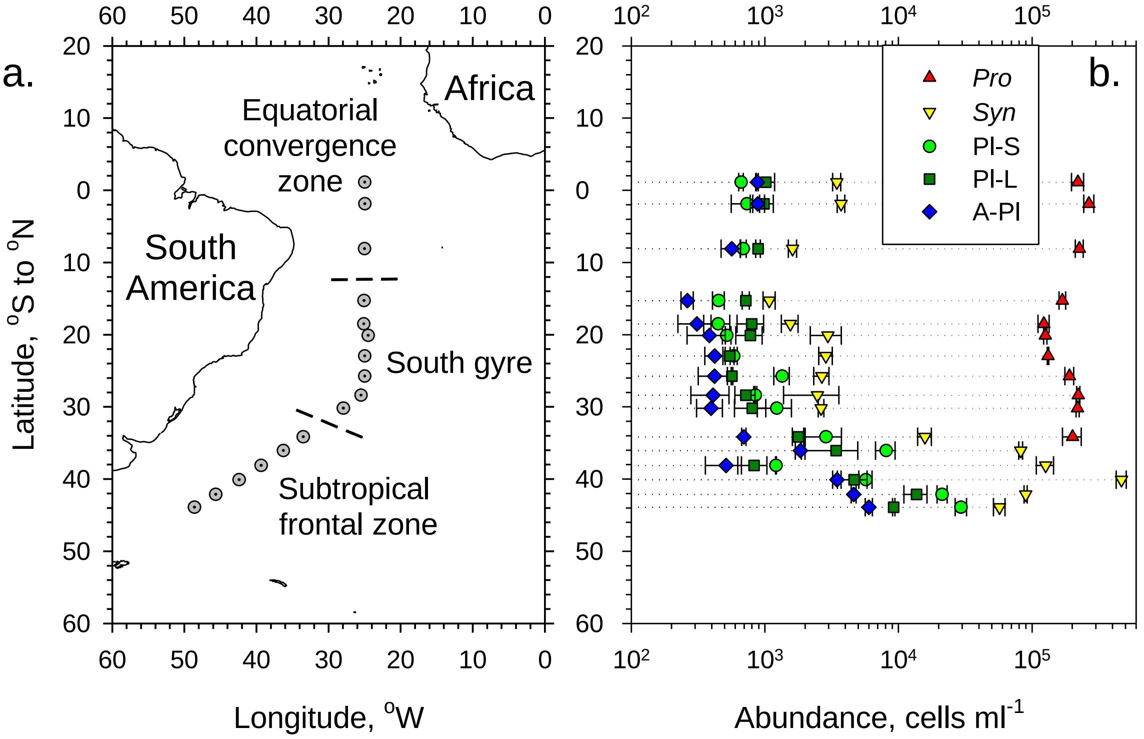

Supplementary Figure 1. Microbial abundances in the studied area. (**a**) A schematic map of the South Atlantic Ocean showing the sampled area. (**b**) Corresponding dot plots of latitudinal distribution of *Prochlororoccus* (*Pro*) and *Synechococcus* (*Syn*) cyanobacteria and small plastidic protists (Pl-S), large plastidic protists (Pl-L) and aplastidic protists (A-Pl). *Syn* and *Pro* distributions were used to identify the boundaries of the three oceanic regions: Equatorial waters (EQ), Southern subtropical gyre (SG) and Southern temperate waters (ST). Short dashed lines (**a**) indicate these boundaries. Circles (**a**) indicate the sampled stations. Error bars (**b**) indicate single standard deviations.

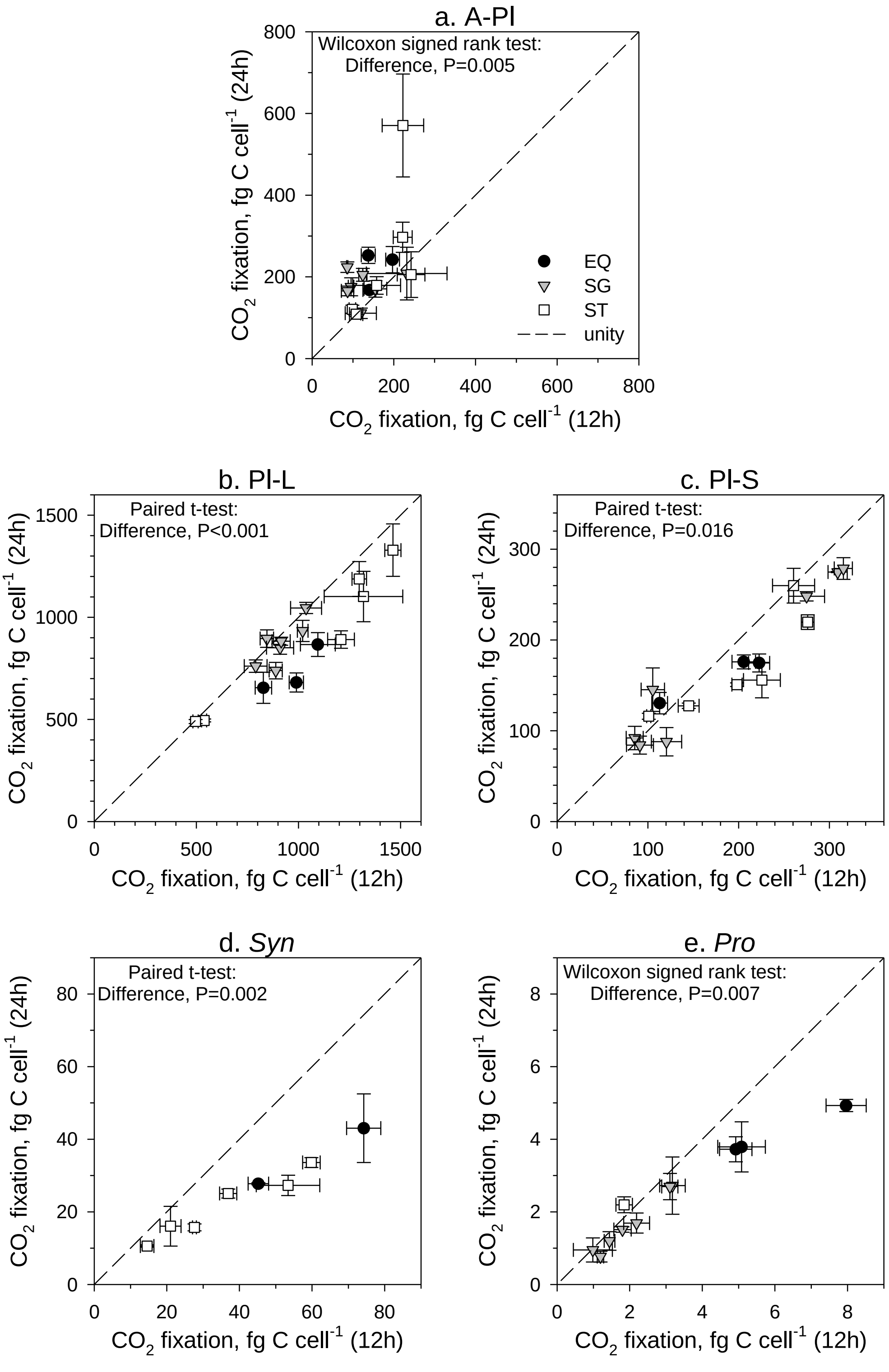

Supplementary Figure 2. Microbial cellular CO_2 fixation. Scatter plot comparison of cellular CO_2 fixation after light incubation (12 h) and 12h light followed by 12 h dark incubation (24 h) of the flow sorted microbial groups: (**a**) aplastidic protists (A-Pl); (**b**) large plastidic protists (Pl-L); (**c**) small plastidic protists (Pl-S); (**d**) *Synechococcus* (*Syn*); (**e**)

Prochlorococcus (*Pro*). The three sampled regions: Equatorial waters (EQ), Southern gyre (SG) and Southern temperate waters (ST). Dashed lines indicate the unity line. Error bars indicate single standard deviation of measurements. The results of Wilcoxon signed rank test in cases when normality test failed ($P<0.05$) or alternatively the results of Paired t-test of 12 h and 24 h measurements with corresponding probabilities (P) are shown at the top of the scatter plots.

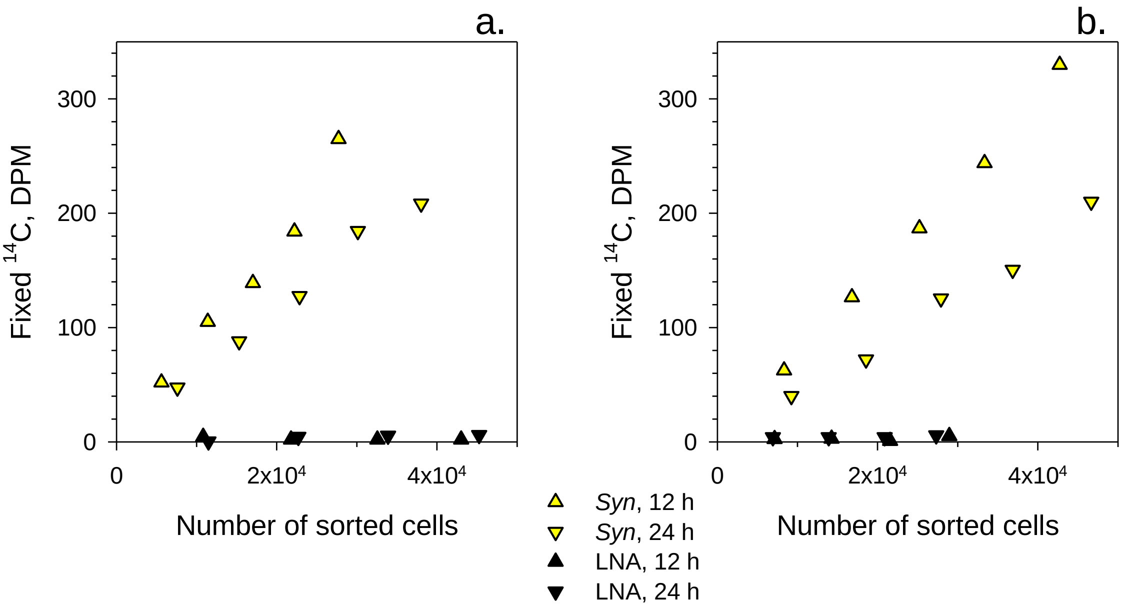

Supplementary Figure 3. Examples of flow sorting results. Comparison of $^{14}CO_2$ fixation after light incubation (12 h) and 12h light followed by 12 h dark incubation (24 h) by different number of flow sorted cells of bacterioplankton with low nucleic acid cellular content (LNA) and *Synechococcus* (*Syn*) at the two stations in the Southern temperate waters: (**a**) 38°6.6’ S, 39°19.8’W and (**b**) 40°6’ S, 42°22.8’W, respectively. Fixation of ^{14}C was radio-assayed as disintegrations per minute (DPM).

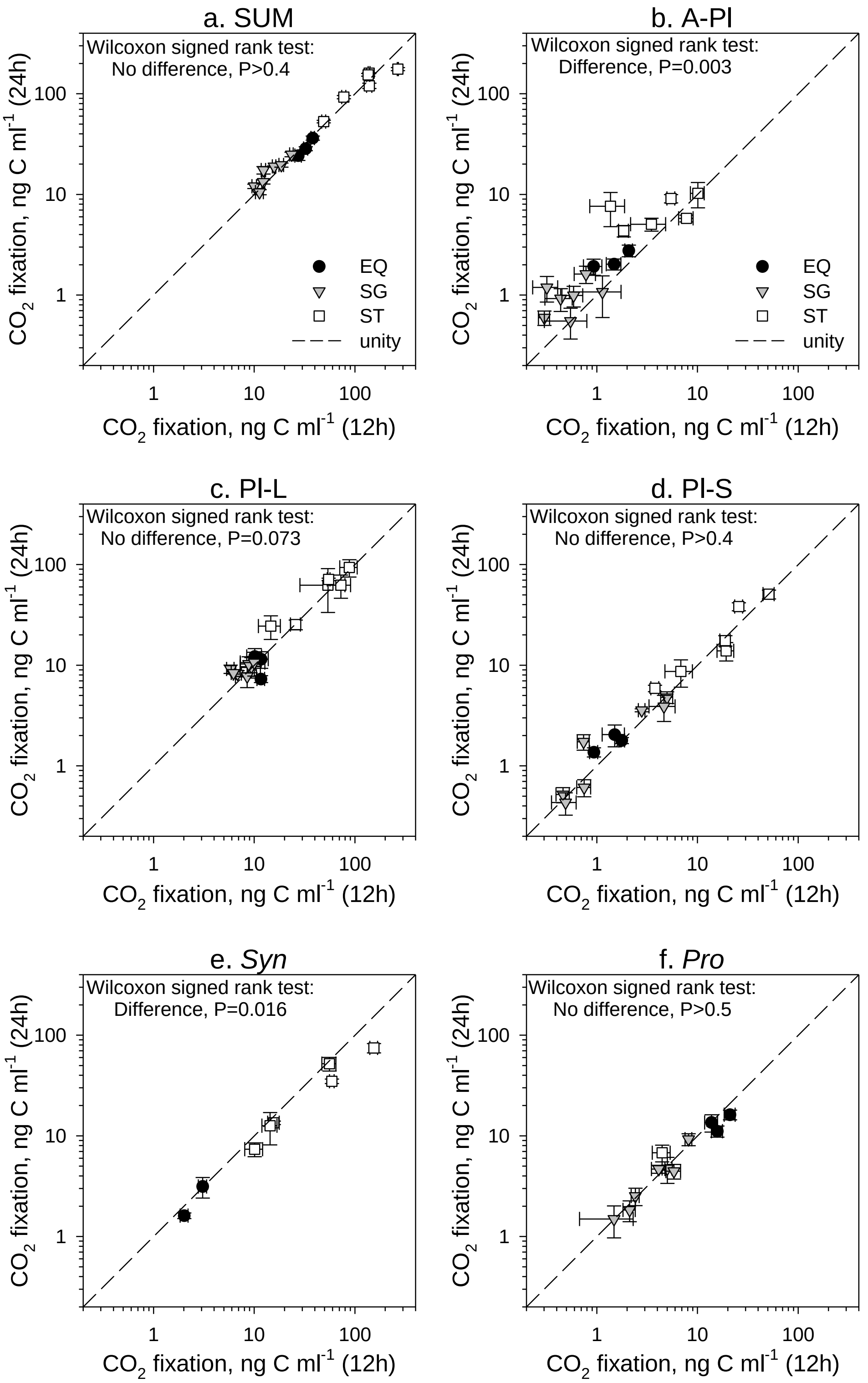

Supplementary Figure 4. CO_2 fixation by microbial populations. Scatter plot comparison of population CO_2 fixation after light incubation (12 h) and 12h light followed by 12 h dark incubation (24 h) of the flow sorted groups: (**a**) the sum (SUM) of all flow sorted groups; (**b**)

aplastidic protists (A-Pl); (**c**) large plastidic protists (Pl-L); (**d**) small plastidic protists (Pl-S); (**e**) *Synechococcus* (*Syn*); (**f**) *Prochlorococcus* (*Pro*). The three studied regions: Equatorial waters (EQ), Southern gyre (SG) and Southern temperate waters (ST). Dashed lines indicate the unity line. Error bars indicate single propagated standard errors. The results of Wilcoxon signed rank test after normality test failed ($P<0.05$) of 12 h and 24 h measurements with corresponding probabilities (P) are shown at the top of the scatter plots.

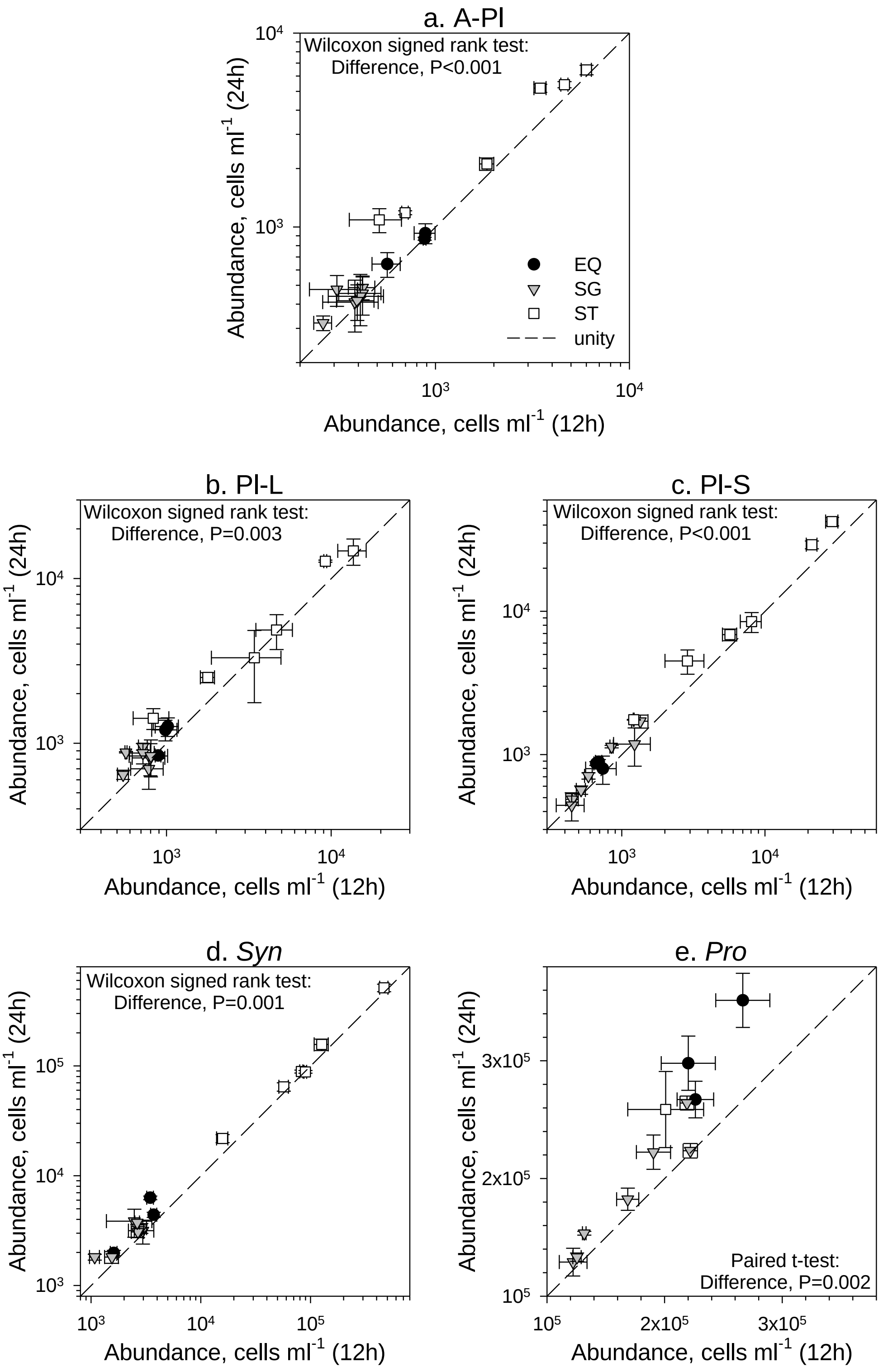

Supplementary Figure 5. Microbial concentrations in CO_2 fixation experiments. Scatter plot comparison of cell abundance after light incubation (12 h) and 12h light followed by 12 h dark incubation (24 h) of the flow sorted groups: (**a**) aplastidic protists (A-Pl); (**b**) large

plastidic protists (Pl-L); (**c**) small plastidic protists (Pl-S); (**d**) *Synechococcus* (*Syn*); (**e**) *Prochlorococcus* (*Pro*). The three studied regions: Equatorial waters (EQ), Southern gyre (SG) and Southern temperate waters (ST). Dashed lines indicate the unity line. Error bars indicate single standard deviation of measurements. The results of Wilcoxon signed rank test in cases when normality test failed ($P<0.05$) or alternatively the results of Paired t-test of 12 h and 24 h measurements with corresponding probabilities (P) are shown at the top or bottom of the scatter plots.

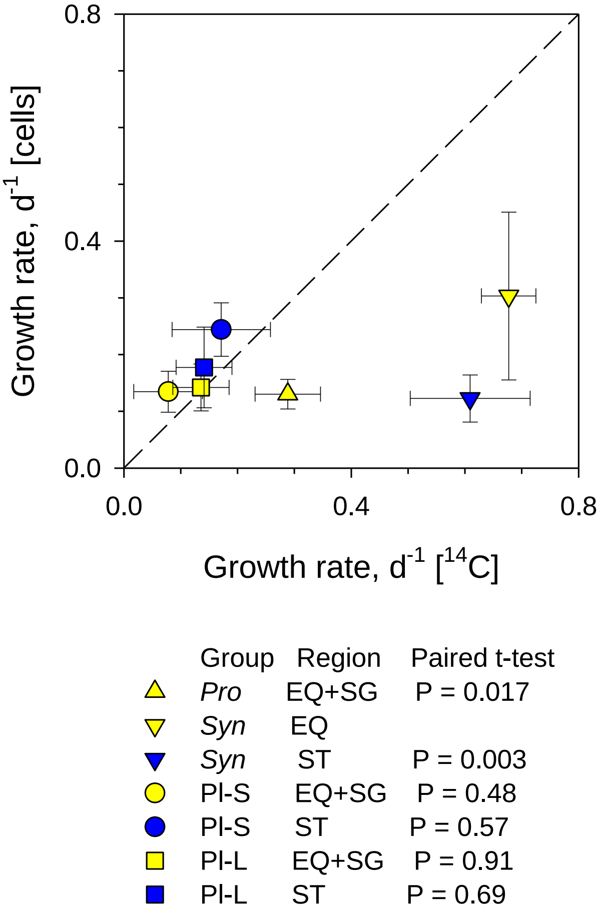

Supplementary Figure 6. Microbial growth rates determined using two methods. Comparison of the mean regional growth rates of *Prochlorococcus* (*Pro*), *Synechococcus* (*Syn*), small plastidic protists (Pl-S) and large plastidic protists (Pl-L) in the two regions: combined Equatorial waters (EQ) and Southern gyre (SG) and Southern temperate waters (ST), assessed using the ^{14}C tracer method and the cell concentration method. Error bars indicate propagated single standard errors. The results of paired t-test are given in the symbol legend. Probability (P) values above 0.05 indicate insignificant differences.
